# Teachers’ Responses to Bullying Questionnaire: A Validation Study in Two Educational Contexts

**DOI:** 10.3389/fpsyg.2022.830850

**Published:** 2022-03-09

**Authors:** Fleur Elisabeth van Gils, Hilde Colpin, Karine Verschueren, Karlien Demol, Isabel Maria ten Bokkel, Ersilia Menesini, Benedetta Emanuela Palladino

**Affiliations:** ^1^School Psychology and Development in Context, Faculty of Psychology and Educational Sciences, KU Leuven, Leuven, Belgium; ^2^Department of Education, Languages, Interculture, Literature and Psychology, University of Florence, Florence, Italy

**Keywords:** bullying, teachers’ responses, CFA, cross-country invariance, elementary education

## Abstract

Given the high prevalence and dramatic impact of being bullied at school, it is crucial to get more insight into how teachers can reduce bullying. So far, few instruments have measured elementary teachers’ responses to bullying. This study investigated the validity of the student-reported Teachers’ Responses to Bullying Questionnaire. The factor structure and measurement invariance were tested across two educational contexts among fourth and fifth grade students from Italy (*n* = 235) and Belgium (*n* = 667). Furthermore, associations between student-perceived teachers’ responses and students’ bullying behavior were examined. Confirmatory Factor Analysis supported the predicted five-factor structure, distinguishing Non-Intervention, Disciplinary Methods, Group Discussion, Mediation, and Victim Support. A partial factor means invariance model was found, allowing for valid comparisons between the Italian and Belgian educational contexts. Significant associations were found between self-reported, but not peer-nominated, bullying behavior and most student-perceived teachers’ responses.

## Introduction

Bullying, commonly defined as repeated and intentional aggressive behavior characterized by an imbalance in power (Olweus, 1993), is widespread in schools. The large-scale Health Behavior in School-aged Children (HBSC) survey in Europe and Canada reported that, on average, 10% of youth had been bullied at school at least two to three times in the last couple of months (Inchley et al., 2020). Bullying is a major problem, because of its detrimental short- and long-term consequences for victimized students (Arseneault, 2018). Given its dramatic impact, it is crucial to reduce and prevent bullying at school.

As the responsible adults in class, teachers may play a key role in tackling bullying incidents among students (Brendgen and Troop-Gordon, 2015; De Luca et al., 2019) and research has shown that teachers use various strategies to do so (e.g., Burger et al., 2015; Wachs et al., 2019). However, research regarding teachers’ strategies to intervene in bullying most often assesses intended responses to hypothetical bullying incidents (e.g., Yoon and Kerber, 2003; Begotti et al., 2017; Colpin et al., 2021). A few studies investigating teachers’ responses to actual bullying incidents are based on teachers’ self-reports (Troop-Gordon and Ladd, 2015; ten Bokkel et al., 2020), but these studies often are qualitative (e.g., Tucker and Maunder, 2015; Acquadro Maran et al., 2017). Teachers’ perceptions of their responses to bullying may be subject to social desirability, but students’ perceptions of teachers’ responses to bullying have largely been neglected until recently (Campaert et al., 2017). Getting insight into students’ perspectives is important, as research demonstrated that student-perceived teacher efforts to reduce bullying were negatively linked to their own bullying behaviors over time (Veenstra et al., 2014).

This study aims at contributing to this gap by investigating the validity of the student-reported Teachers’ Responses to Bullying Questionnaire (TRBQ; Campaert et al., 2017; Nappa et al., 2021) in a sample of fourth and fifth grade elementary school students. More specifically, a cross-country validation in two European countries was conducted to ensure the suitability of the questionnaire in different educational contexts. Interpretations and perceptions of social behaviors in schools, including teachers’ responses to bullying, may differ across educational and cultural contexts. Although the duration and design of elementary education in European countries is very similar (EURYDICE, 2018), some differences can be noted. Whereas schools in Italy should appoint a responsible teacher for (cyber) bullying and a free online training on bullying prevention is provided by the Ministry of Education, in Flanders (Belgium) no specific governmental regulations are in place nor teacher training regarding the topic of bullying. Findings can only be generalized and compared when the instrument measures student-perceived teachers’ responses similarly across contexts, which is key in further advancing this research domain.

### Teachers’ Responses to Bullying

Bullying is considered a complex group process (Salmivalli et al., 1996), which takes place in a broader social-ecological system. Consequently, not only individual characteristics of students affect bullying processes, but also the interplay between the individual and his or her environment (Hong and Espelage, 2012). Bullying often takes place within a school environment, with teachers as key interaction partners. Only recently, the role of teachers in bullying processes has been studied more extensively (Gest and Rodkin, 2011; Brendgen and Troop-Gordon, 2015). This research has shown that teachers can respond in various ways to bullying incidents among students (e.g., Bauman et al., 2008; Burger et al., 2015; Troop-Gordon and Ladd, 2015; Wachs et al., 2019).

Although scholars have attempted to categorize these teachers’ responses, until today no clear and common categorization is used in research regarding teachers’ responses to bullying (e.g., Colpin et al., 2021). However, some recurring themes within these categorizations can be identified. First, a distinction between active and passive responses can be made. Active responses are direct efforts to avert further victimization (Troop-Gordon and Quenette, 2010), such as verbally reprimanding the student who bullied and helping the involved students to find a solution for the bullying incident (Burger et al., 2015; Campaert et al., 2017). Regarding passive responses, for instance, a teacher may not notice, not respond to or ignore a bullying situation (e.g., Bauman et al., 2008; Rigby, 2014; Yoon et al., 2016; Campaert et al., 2017), or a teacher may focus on the victimized student’s own coping with the bullying (Troop-Gordon and Quenette, 2010). Second, the responses can be either solely focused on individuals, such as the student who bullied or the victimized student, or involve a larger group, such as the whole class. Examples of responses focused on individuals are supporting the victimized student (Campaert et al., 2017) and disciplining the student who bullied (Burger et al., 2015; Troop-Gordon et al., 2021). Examples of responses addressed toward a group are discussing rules with the whole class (Yoon and Kerber, 2003) and supporting positive relationships among students (Wachs et al., 2019). In addition, some researchers acknowledge the role of others, such as colleagues of the teacher, the head of the school, or parents, in responding to the bullying situation (e.g., Bauman et al., 2008; Wachs et al., 2019; Rigby, 2020). Third, a more traditional authoritarian punishment approach can be distinguished from a non-punitive, restorative approach (Bauman et al., 2008; Rigby, 2014; Burger et al., 2015). Examples of a punitive approach are reprimands and sanctions, while a non-punitive approach is characterized by working with students who bullied, such as increasing their empathy and giving them insight into the harm of their bullying behavior for the victimized student.

In line with the lack of a clear categorization of teachers’ responses to bullying so far, also valid and reliable instruments to measure teachers’ responses are scarce and further measurement development is required (Bauman et al., 2021; Troop-Gordon et al., 2021). Most currently available instruments measure teachers’ responses to hypothetical bullying scenarios, such as the Handling Bullying Questionnaire (Bauman et al., 2008). However, these scenarios assess what teachers think they would do, which can be different from their actual behavior in practice. In addition, the scenarios do not necessarily represent the complexity of real-life bullying situations (Wachs et al., 2019; Fischer et al., 2020). Therefore, measuring teachers’ responses to actual bullying has gained more attention over the past few years (e.g., Troop-Gordon and Ladd, 2015). Only recently, some instruments have been developed which measure the invaluable student perspective of teachers’ responses to actual bullying (Wachs et al., 2019; Rigby, 2020; Troop-Gordon et al., 2021).

The present study aimed to contribute to this emerging field of research by investigating an instrument assessing teachers’ responses to actual bullying as perceived by students (Campaert et al., 2017; Nappa et al., 2021). The original instrument, developed by Campaert et al. (2017), distinguished teachers’ interventions and non-intervention toward perpetrators (11 items) from teachers’ interventions and non-intervention toward victims of bullying (11 items). Confirmatory Factor Analysis (CFA) revealed a well-fitting factor structure with the factors Group Discussion, Disciplinary Sanctions, Mediation, and Non-Intervention within the bullying scale and the factors Group Discussion, Victim Support, Mediation, and Non-Intervention within the victimization scale. Further, internal consistency of the factors ranged from minimally acceptable to respectable (DeVellis, 1991) in a sample of 609 fifth and seventh grade Italian students. Measurement invariance analyses confirmed partial scalar invariance across grade level for both bullying and victimization responses. A positive indirect effect of Non-Intervention on bullying was found, whereas Disciplinary Sanctions and Victim Support had a negative indirect effect on bullying. All indirect effects were mediated by moral disengagement.

Nappa et al. (2021) revised the original instrument by omitting the distinction between bullying and victimization. They formed a 15-item questionnaire with five overall subscales of teachers’ responses to bullying with three items each. First, Non-Intervention represents when a teacher does not intervene in a bullying situation, either intentionally or because the teacher is not aware of the bullying situation. Second, Disciplinary Methods implies that a teacher applies sanctions to the student(s) who bullied. Third, Group Discussion means that a teacher involves the whole class or a group of students to discuss bullying situations. Fourth, Mediation implies that a teacher acts informally as an intermediary to give the involved students the opportunity to express their point of view. Fifth, Victim Support means that a teacher provides support to the victimized student(s). In a sample of 1,406 middle and high school students (seventh to ninth grade) from the Italian region Tuscany, evidence was found for a higher-order factor model. This higher-order factor model consisted of Supportive/Relational Interventions as a second-order factor measured by Group Discussion, Mediation, and Victim Support, along with Non-Intervention and Disciplinary Methods as first-order factors. Internal consistencies ranged from minimally acceptable to very good (DeVellis, 1991). The instrument was used to investigate the effects of parents’ and teachers’ responses to offline bulling on cyberbullying. For parents, Supportive/Relational Interventions were associated with lower levels of cyberbullying. For teachers, an association between Non-Intervention and higher levels of cyberbullying was found.

### Current Study

Given the scarcity of reliable instruments to measure teachers’ responses to bullying as perceived by students, more research is needed. Therefore, the aim of the current study was to contribute to this research domain by validating a measure of student-perceived teachers’ responses to bullying in elementary school in two educational contexts. As elementary students typically have one teacher (in Belgium), or a main reference teacher (in Italy), these teachers have a unique position to shape social experiences, including bullying, in their class (e.g., Demol et al., 2020; EURYDICE, 2018). Moreover, in the second half of elementary school, peer relationships become more important for children (e.g., De Laet et al., 2014) and bullying rates are relatively high (Inchley et al., 2020), making this a challenging yet important period for bullying prevention.

We investigated the student-reported Teachers’ Responses to Bullying Questionnaire (TRBQ), which is, in its current form, formerly validated by Nappa et al. (2021) for use in Italian middle and high school samples. Specifically, we examined whether the TRBQ is valid to use among elementary students in the educational contexts of Italy and Belgium. First, the factor structure of the TRBQ in both contexts was evaluated. We expected support for a five-factor structure, based on the *a priori* composition of the questionnaire as developed for and used in the study of Nappa et al. (2021). However, an alternative factor structure was plausible, since Nappa et al. (2021) found high correlations between the Group Discussion, Mediation, and Victim Support scales which led to testing a model with a second order factor (i.e., Supportive/Relational Interventions). Therefore, alternative factor models with two and three factors were examined. The first three-factor model resembled the second-order factor structure found by Nappa et al. (2021) and the second three-factor model represented the factors not intervening, individual interventions, and group interventions. The two-factor model distinguished not intervening from all actual interventions. Second, measurement invariance across the two educational contexts was evaluated in the model with the best-fitting structure. Third, we investigated if the associations between student-perceived teachers’ responses and bullying behavior are the same in the educational contexts, which can contribute to the convergent validity of the TRBQ. Since studies have not been carried out yet on this topic with children at this age, we adopted an explorative approach without specific hypotheses about possible differences across the educational contexts. However, if we would find support for the expected five-factor structure, Non-Intervention was expected to be positively associated with bullying behavior. Furthermore, negative associations between active teachers’ responses and bullying were expected (cf. Campaert et al., 2017; Nappa et al., 2021).

## Materials and Methods

### Participants and Procedure

The participants in this study were part of two independent samples in Italy and Belgium. All students in grades 4 and 5 of the recruited schools were eligible for participating in the study. The Italian sample consisted of 271 students (44% girls) in grades 4 and 5 (12 classes) of seven elementary schools in the region Tuscany (response rate = 94%). The students completed the questionnaire in December 2019, before their school started to participate in the KiVa program. Data were collected with paper-and-pencil questionnaires, supervised by a KiVa trainer (i.e., both a psychologist and a trained research assistant). In accordance with the Italian law on the protection of minors, preliminary informed consent, consisting of initial approval by the school principal and class council, was requested. Once permission was gained from the schools, active informed consent was obtained from the students’ parents.

The Belgian sample was part of a three-wave longitudinal study in grades 4 to 6 (62 classes) of 13 elementary schools (*N* = 1,051, response rate = 81%) in the Dutch-speaking region Flanders. Data were collected with paper-and-pencil questionnaires, supervised by a researcher, at the beginning, middle, and end of school year 2018-2019. Ethical approval from the institutional review board was obtained for this study and active informed consent was obtained from the students’ parents. To make a balanced comparison with the Italian sample, a subsample from the original dataset was extracted, consisting of all students in grade 4 and 5 (43 classes, *n* = 688, *M*_age_ = 9.58 years, *SD*_age_ = 0.67, 53% girls, response rate = 79%). This subsample was used for the present study and will be further referred to as the Belgian sample. Most of these students were born in Belgium (90%). Other birth countries were the Netherlands (1.5%), Poland (0.4%), Turkey (0.3%), and Morocco (0.3%). The data of the first wave were used, since these were collected in November 2018, a similar time period within the school year as in which the Italian data were collected.

Hence, the total sample in the present study consisted of 959 Italian and Belgian students in 55 classes within 20 schools. Due to absence (e.g., illness) at the time of administration, 32 students (i.e., 14 Italian and 18 Belgian students) did not participate. Moreover, 25 students (i.e., 22 Italian and 3 Belgian students) were also excluded, because they did not answer any of the TRBQ items. Therefore, our total sample for the main analyses consisted of 902 students (i.e., 235 Italian and 667 Belgian students, 51% girls) in grades 4 (45%) and 5 (55%). Since the imbalance in Italian and Belgian sample size could lead to incorrect conclusions about measurement invariance, simulated datasets with balanced sample sizes (Yoon and Lai, 2018) were used in the analyses with the total sample (see Data Analyses).

### Measures

The questionnaires were introduced by a description of bullying in order to minimize the influence of subjective interpretations. The description was based on the definition of Olweus (1993), emphasizing the key elements of bullying (i.e., intentional, repetitive, and power imbalance), including examples, and differentiating teasing from bullying. Students could reread the description at any time during the administration.

#### Teachers’ Responses to Bullying Incidents

To assess students’ perceptions of their teachers’ responses to bullying incidents, the Teachers’ Responses to Bullying Questionnaire (TRBQ; Nappa et al., 2021) was administered. This questionnaire is a revision of the measure presented in the work of Campaert et al. (2017) and it is intended to assess students’ perspectives of five possible teachers’ responses to bullying incidents among students. Accordingly, the TRBQ consists of five scales: Non-Intervention, Disciplinary Methods, Group Discussion, Mediation, and Victim Support. Each scale consists of three items, resulting in a 15-item questionnaire (see Appendix). Students rated the items on a 5-point Likert scale (1 = *Never*, 2 = *Almost never*, 3 = *Sometimes*, 4 = *Often*, 5 = *Always*). In the Italian questionnaire, a written explanation of the answer options was added to the Non-Intervention items to help students understand the meaning of the different answers. In the Belgian questionnaire, no written explanation was added. However, additional verbal instructions on the double negotiation were given after answering difficulties were discovered during data collection.

To develop a Dutch version of the TRBQ, a translation and backtranslation process based on the English version of the original questionnaire was performed (Campaert et al., 2017). Specifically, the English version was translated into Dutch by a first researcher. A second researcher translated the questionnaire back to English, and these translations were matched by a third researcher. Afterward, the translation was checked against the original Italian items of Nappa et al. (2021) with help of a Belgian researcher who speaks Italian fluently and some last adaptations to the wording were done.

#### Bullying Behavior

To investigate which students bullied other students, a multi-informant approach was used. Hence, both self-reported and peer-nominated measures were used.

##### Self-Reported Bullying Behavior

The students were asked to rate how often they had bullied another student during the past months on a 5-point Likert scale from 1 (*I have not bullied*) to 5 (*Several times a week*). This item originates from the validated Olweus Bullying Questionnaire - Revised (Solberg and Olweus, 2003).

##### Peer-Nominated Bullying Behavior

Peers nominated classmates who, in their opinion, bullied other students by answering the question ‘Which boys/girls take the initiative to bully classmates? (Italian sample; Menesini and Gini, 2000) and ‘Which classmates bully other students at school?’ (Belgian sample; Salmivalli et al., 1996; Salmivalli and Voeten, 2004). They were allowed to nominate an unlimited number of classmates (Marks et al., 2013). Students of the Italian sample were allowed to also nominate themselves, whereas Belgian students could not nominate themselves. To form standardized proportion scores of bullying within classrooms, the number of nominations a student received was summed and divided by the number of students who completed the questionnaire (van den Berg et al., 2015). For the Belgian sample, the number of students who completed a questionnaire minus one was used, as self-nominations were not included in the peer nomination scores. To ensure a reliable and valid nomination procedure, peer-nominated bullying measures were not taken into account in the analyses for classes in which less than 60% of the students participated (Cillessen, 2009). In addition, the measures of classes with less than 10 students were not included in the analyses.

#### Data Analyses

Analyses were conducted in Mplus (Version 8.4). We analyzed the data using Structural Equation Modeling (SEM). More specifically, CFA was used to examine the factor structure. After obtaining the best-fitting model, the internal consistency of the factors was examined. Second, we tested for measurement invariance of the TRBQ across Italian and Belgian students. Third, we examined whether the associations between student-perceived teachers’ responses to bullying and students’ bullying behavior differed for the Italian and Belgian sample. To take into account the missing data (Italian sample: 0.9-27.2%; Belgian sample: 0.1-1.9%), Full Information Maximum Likelihood (FIML) was used in the analyses. The high amount of missingness can be attributed to a large number of Italian students (i.e., 63) who did not complete any items of the Non-Intervention scale. Due to a non-normal distribution of the TRBQ items and both bullying measures, Robust Maximum Likelihood estimation was used in the analyses.

First, the factor structure was tested in three samples: the Italian sample, the Belgian sample, and the total sample. As the Belgian sample was larger than the Italian sample and fit indices are substantially affected by sample size (Marsh et al., 1988), simulated datasets with balanced samples (Yoon and Lai, 2018) were used for analyses with the total sample. It is essential to apply such a technique, since groups with larger samples have more weight in determining the final solution (Yoon and Lai, 2018). Using simulated datasets with balanced samples implies that we selected separate datasets from the total sample 100 times, with all participants of the smaller Italian group (i.e., all 235 Italian participants) and a randomly selected subset of the participants of the larger Belgian group (i.e., a subset of 235 Belgian participants). Consequently, 100 different simulated datasets with an average sample of 470 participants were used to conduct the CFAs, resulting in mean fit indices across the 100 replications. In order to detect the best-fitting model, we compared four models (i.e., Model A to D). Model A, a five-factor model, represented the original structure of the questionnaire with latent constructs for all scales: Non-Intervention, Disciplinary Methods, Group Discussion, Mediation, and Victim Support. Further, two different three-factor models were tested. In the first three-factor model, Model B, the three latent constructs represented not intervening (i.e., scale Non-Intervention), supportive/relational interventions (i.e., scales Group Discussion, Mediation, and Victim Support), and authoritarian interventions (i.e., scale Disciplinary Methods). This three-factor model resembled the structure of the second-order factor model as found by Nappa et al. (2021). The second three-factor model, Model C, consisted of the latent constructs not intervening (i.e., scale Non-Intervention), individual bully and/or victim interventions (i.e., scales Disciplinary Methods, Mediation, and Victim Support), and class group interventions (i.e., scale Group Discussion). Finally, Model D, a two-factor model, represented two separate latent constructs for not intervening (i.e., scale Non-Intervention) and all actual interventions (i.e., scales Disciplinary Methods, Group Discussion, Mediation, and Victim Support).

Second, the best-fitting model found in the first set of analyses was used to conduct measurement invariance analyses. Measurement invariance was examined by using stepwise multigroup CFAs to investigate whether the TRBQ allows for valid comparisons between individuals from the Italian and Belgian sample. Nested models were compared from least constrained (Model 0) to most restrictive (Model 5) by placing equality constraints on the parameters across the Italian and Belgian educational contexts. Often-used stepwise constraints are accommodated in configural, weak, strong, and strict factorial invariance models (Meredith and Teresi, 2006; Marsh et al., 2010). First, all parameters were estimated freely across countries (Model 0; configural invariance). This model tested whether the factor structure showed an adequate fit for both the Italian and Belgian educational contexts and provided a baseline model for comparing the following constrained models. Second, factor loadings were constrained to be equal across countries (Model 1; weak factorial invariance). The invariance of intercepts was tested in a third step (Model 2; strong factorial invariance). Fourth, residual variances were constrained to be equal across countries (Model 3; strict factorial invariance). After carrying out the four steps to investigate measurement invariance, we examined possible group differences in the latent constructs by performing step five and six (Marsh et al., 2010). Step five was carried out by placing an equality constraint on the factor (co)variances (Model 4; factor (co)variance invariance). Lastly, factor means were constrained to be equal across countries (Model 5; factor mean invariance). In this way, we could detect which latent means differ between the two educational contexts.

Third, we investigated the associations between student-perceived teachers’ responses and both bullying measures. In two separate models, we respectively added correlations between student-perceived teachers’ responses and students’ self-reported and peer-nominated bullying behavior to the final measurement invariance model. To test for invariance of the associations in the two samples, a model with freely estimated associations across the Italian and Belgian sample was compared with a model in which the associations were restricted to be the same across the samples. If the restricted model does not fit the data worse than the freely estimated model, the associations between student-perceived teachers’ responses and bullying can be assumed the same across the Italian and Belgian sample. To conduct the analyses with the self-reported bullying measure the same cluster of 100 datasets was used. However, a new set of 100 datasets, in which nine Belgian classes were excluded (*n* = 92), was created to conduct the analyses with the peer-nominated bullying measure. These classes (i.e., three classes with less than 10 students participating and six classes with less than 60% of the students participating) were excluded from the sample. Since no Italian classes had to be excluded, the number of students from each sample in the balanced datasets remained 235. As simulated datasets were used, the significance of the associations was evaluated across 100 datasets. Therefore, the percentage of significant coefficients was reported, which represents the percentage of coefficients across the datasets that were significant.

In all analyses, model fit was evaluated with standard model fit indices (Hu and Bentler, 1999): the Comparative Fit Index (CFI; should exceed 0.90 and preferably 0.95), the Root Mean Square Error of Approximation (RMSEA; should be smaller than 0.08, preferably less than 0.05), the Standardized Root Mean Square Residual (SRMR; should be smaller than 0.08), and the Bayesian Information Criterion (BIC; with lower values indicating better models). Although different models usually are compared by chi-square difference testing (Geiser, 2012), this was not possible in our study since simulated datasets were used. Moreover, chi-square values are affected by sample size. Especially large samples may lead to a rejection of the model based on chi-square testing (Kline, 2015). Therefore, chi-square values were reported, but were not used for difference testing. Instead, the different models were compared by evaluating the change in CFI, RMSEA, and BIC. Chen (2007) suggests changes of <0.010 in CFI and <0.015 in RMSEA to have support for the more parsimonious model. We aimed to meet the guidelines of all three fit indices. The invariance models were compared sequentially by testing the decrease in model fit. Invariance holds when the more parsimonious model fits the data equally well as the more general model. If the change in CFI and RMSEA was too large to find support for the more parsimonious model, we explored which parameter failed the test of invariance to release this parameter and obtain partial measurement invariance. Since no modification indices are reported in mean results over various simulated datasets, modification indices of the individual simulated datasets were examined. A parameter that clearly emerged as a parameter to improve the model in preferably half (or more) of the datasets or at least in 25% of the datasets, was estimated freely in the analyses with 100 simulated datasets to attain partial invariance.

## Results

### Factor Structure

Confirmatory Factor Analysis were used to examine the factor structure of the TRBQ in the Italian, Belgian, and total sample. Fit indices of the four models tested are shown in Table 1. Model A, the five-factor model, yielded a good fit in all samples (i.e., total sample: CFI = 0.965, RMSEA = 0.038, SRMR = 0.045; Italian sample: CFI = 0.974, RMSEA = 0.036, SRMR = 0.052; Belgian sample: CFI = 0.940, RMSEA = 0.047, SRMR = 0.046) and showed a better fit compared with all other models. Therefore, the five-factor structure of the TRBQ was confirmed.

**TABLE 1:** Confirmatory factor analyses fit indices for models with five to two factors for total, Italian, and Belgian sample.

Model & sample		χ^2^	Df	*p*	CFI	ΔCFI	RMSEA	ΔRMSEA	SRMR	BIC	ΔBIC
A. Five-factor ^a, b^		135.37	80		0.965		0.038		0.045	20,360	
	Italian	104.92	80	0.032	0.974		0.036		0.052	9,678	
	Belgian	199.68	80	< 0.001	0.940		0.047		0.046	30,030	
B. Three-factor ^a,c^		305.87	87		0.861	−0.104	0.073	+ 0.035	0.063	20,530	+ 170
	Italian	177.47	87	< 0.001	0.905	−0.069	0.067	+ 0.031	0.063	9,727	+ 49
	Belgian	462.12	87	< 0.001	0.812	−0.128	0.080	+ 0.033	0.067	30,320	+ 290
C.Three-factor ^a,d^		215.20	87		0.919	−0.046	0.056	+ 0.018	0.053	20,420	+ 60
	Italian	174.93	87	< 0.001	0.908	−0.066	0.066	+ 0.030	0.061	9,727	+ 49
	Belgian	282.49	87	< 0.001	0.902	−0.038	0.058	+ 0.011	0.053	30,093	+ 63
D. Two-factor ^a,e^		350.46	89		0.834	−0.131	0.079	+ 0.041	0.066	20,578	+ 218
	Italian	209.86	89	< 0.001	0.874	−0.100	0.076	+ 0.040	0.066	9,758	+ 80
	Belgian	514.78	89	< 0.001	0.787	−0.153	0.085	+ 0.038	0.069	30,375	+ 345

*N_total sample_, = 470; n_Italian sample_ = 235; n_Belgian sample_ = 667. CFI, Comparative fit index; RMSEA, Root mean square error of approximation; SRMR, Standardized root mean square residual; BIC, Bayesian information criterion. CFI, RMSEA, and BIC differences are relative to the five-factor baseline model and between the respective samples. ^a^ These model indicators represent the fit indices of the total sample. ^b^ Model A consisted of five factors: Non-Intervention, Disciplinary Methods, Group Discussion, Mediation, and Victim Support. ^c^ Model B consisted of three factors: not intervening (i.e., scale Non-Intervention), supportive/relational interventions (i.e., scales Group Discussion, Mediation, and Victim Support), and authoritarian interventions (i.e., scale Disciplinary Methods). ^d^ Model C consisted of three factors: not intervening (i.e., scale Non-Intervention), individual bully and/or victim interventions (i.e., scales Disciplinary Methods, Mediation, and Victim Support), and class group interventions (i.e., scale Group Discussion). ^e^ Model D consisted of two factors: not intervening (i.e., scale Non-Intervention) and all actual interventions (i.e., scales Disciplinary Methods, Group Discussion, Mediation, and Victim Support).*

The internal consistency of the factors in the five-factor model ranged from unacceptable to very good (DeVellis, 1991). Cronbach’s alphas in the Italian and Belgian sample respectively were 0.61 and 0.52 (Non-Intervention), 0.71 and 0.65 (Disciplinary methods), 0.71 and 0.76 (Group Discussion), 0.75 and 0.62 (Mediation), and 0.84 and 0.76 (Victim Support).

### Measurement Invariance

To test whether the five-factor 15-indicator structure of student-perceived teachers’ responses to bullying was the same across Italian and Belgian students, we first estimated the configural invariance model (Model 0). Fit indices and changes in fit indices of the different models tested are shown in Table 2. The adequate model fit of the configural invariance model indicated that the factor structure was similar across both the Italian and Belgian educational contexts.

**TABLE 2:** Measurement invariance models comparing the Italian and Belgian sample.

	Model	Compared model	χ^2^	df	CFI	ΔCFI	RMSEA	ΔRMSEA	SRMR	BIC	ΔBIC
0	Configural inv.		235.02	160	0.956		0.044		0.054	20,456	
1	Weak factorial inv. ^a^	0	248.31	170	0.954	−0.002	0.044	0.000	0.062	20,413	−43
2	Strong factorial inv. ^b^	1	295.53	180	0.932	−0.022	0.052	+ 0.008	0.070	20,405	−8
2.1	Partial strong factorial inv. ^c^	1	277.97	179	0.941	−0.013	0.048	+ 0.004	0.067	20,391	−22
3	Strict factorial inv. ^d^	2.1	321.96	194	0.924	−0.017	0.053	+ 0.005	0.083	20,355	−36
3.1	Partial strict factorial inv. ^e^	2.1	313.55	193	0.929	−0.012	0.051	+ 0.003	0.079	20,351	−40
3.2	Partial strict factorial inv. ^f^	2.1	304.13	192	0.934	−0.007	0.050	+ 0.002	0.078	20,346	−45
4	Factor (co)var. inv. ^g^	3.2	345.97	207	0.918	−0.016	0.053	+ 0.003	0.103	20,309	−37
4.1	Partial factor (co)var. inv. ^h^	3.2	333.58	206	0.924	−0.010	0.051	+ 0.001	0.099	20,299	−47
5	Factor means inv. ^i^	4.1	385.40	211	0.897	−0.027	0.059	+ 0.008	0.113	20,329	+ 30
5.1	Partial factor means inv. ^j^	4.1	342.47	210	0.922	−0.002	0.052	+ 0.001	0.103	20,284	−15

*CFI, Comparative fit index; RMSEA, Root mean square error of approximation; SRMR, Standardized root mean square residual; BIC, Bayesian information criterion; inv., invariance; (co)var., (co)variance.*

*^a^ Model with constrained factor loadings across countries. ^b^ Model with constrained intercepts and free latent means on top of the constraints of Model 1. ^c^ Model as Model 2, with the intercept of item 9 released, so it could vary across countries. ^d^ Model with constrained residual variances on top of the constraints of Model 2.1. ^e^ Model as Model 3, with the residual variance of item 7 released.^f^ Model as Model 3.1, with the residual variance of item 2 released. ^g^ Model with constrained factor (co)variances on top of the constraints of Model 3.2. ^h^ Model as Model 4, with the factor variance of Non-Intervention released. ^i^ Model with constrained factor means on top of the constraints of Model 4.1. ^j^ Model as Model 5, with the factor mean of Group Discussion released.*

Second, Model 0 was compared with a weak factorial invariance model (Model 1) in which the factor loadings were constrained to be equal across countries. All changes in fit indices indicated that the invariance of factor loadings did not result in a worse fit compared with Model 0 (|ΔCFI| = 0.002, |ΔRMSEA| = 0.000, and ΔBIC = −43). Thus, support for weak factorial invariance was found.

Third, strong factorial measurement invariance was tested by adding constraints of equal intercepts and free latent means on top of the constraints of Model 1. The change in RMSEA showed support for Model 2, whereas the change in CFI did not and only a small decrease in BIC was found (|ΔCFI| = 0.022, |ΔRMSEA| = 0.008, and ΔBIC = −8). Overall, Model 2 fitted the data worse than Model 1. Therefore, the modification indices of the individual datasets were examined to find out which intercepts were non-invariant. In 96 out of 100 datasets the intercept of item 9 (see Appendix; “My teacher tries to have the victim consoled and helped by other students in the class,” belonging to the factor Victim Support) showed strong non-invariance. Item 9 had a higher intercept for Italian students. This indicated that, compared with Belgian students, Italian students gave a higher score on this item given the same level of Victim Support. When the intercept of item 9 was allowed to vary across the groups (Model 2.1), changes in the fit indices RMSEA and BIC showed support for this model (|ΔCFI| = 0.013, |ΔRMSEA| = 0.004, and ΔBIC = −22). Furthermore, the change in CFI was not so distant from the cut-off. Since no other parameter clearly emerged to be released, we concluded partial strong factorial invariance holds with this model.

Fourth, Model 2.1 was compared with a strict factorial invariance model (Model 3) in which the residual variances were constrained to be equal across groups on top of the constraints in Model 2.1. The change in CFI did not show support for Model 3, whereas the changes in RMSEA and BIC did (|ΔCFI| = 0.017, |ΔRMSEA| = 0.005, and ΔBIC = −36). Overall, Model 3 fitted the data worse than Model 2.1. Again, the modification indices were inspected. Several items with non-invariance emerged in less than half of the datasets. We chose to free the residual variance of item 7 (“My teacher tries to get the students to make peace”, belonging to the factor Mediation) across countries, since this item was indicated in most datasets (*n* = 30). Item 7 had a higher residual variance for Belgian students, indicating that the factor Mediation explained more variance of item 7 for the Italian students than the Belgian students. When the residual variance of item 7 was allowed to vary across groups (Model 3.1), no adequate change in CFI was obtained (|ΔCFI| = 0.012, |ΔRMSEA| = 0.003, and ΔBIC = −40). Therefore, in addition to item 7, the residual variance of item 2 (“My teacher does not notice the bullying”, belonging to the factor Non-Intervention) was estimated freely across countries (Model 3.2), as it emerged in the modification indices of 27 datasets. Item 2 had a higher residual variance for Belgian students, indicating that the factor Non-Intervention explained more variance of item 2 for the Italian students than the Belgian students. Model 3.2 showed support for partial strict factorial invariance (|ΔCFI| = 0.007, |ΔRMSEA| = 0.002, and ΔBIC = −45).

Fifth, to obtain Model 4, equality constraints for factor (co)variances were added on top of the constraints in Model 3.2. The change in CFI did not show support for Model 4, whereas the changes in RMSEA and BIC did (|ΔCFI| = 0.016, |ΔRMSEA| = 0.003, and ΔBIC = −37). Overall, the model of factor (co)variances invariance (Model 4) showed a worse model fit compared with Model 3.2. Strong non-invariance for the factor variance of Non-Intervention was found in 57 datasets. The factor variance of Non-Intervention was higher for Belgian students, indicating a larger spread of factor scores for the Belgian compared to the Italian students. When allowing the factor variance of Non-Intervention to vary across groups, a partial factor (co)variances invariance model was obtained (Model 4.1; |ΔCFI| = 0.010, |ΔRMSEA| = 0.001, and ΔBIC = −47).

Sixth, Model 4.1 was compared with a model in which factor means were constrained to be equal across groups (Model 5). The change in CFI and BIC did not show support for Model 5, whereas the change in RMSEA did (|ΔCFI| = 0.027, |ΔRMSEA| = 0.008, and ΔBIC = + 30). Overall, Model 5 fitted the data worse than Model 4.1. In all datasets, the mean of Group Discussion had a strong non-invariance. The factor mean of Group Discussion was higher for Italian students, indicating that they had a higher latent mean score for Group Discussion as compared to Belgian students. When the factor mean of Group Discussion was allowed to be freely estimated across countries, a final partial factor means invariance model was obtained (Model 5.1; |ΔCFI| = 0.002, |ΔRMSEA| = 0.001, and ΔBIC = −15).

### Associations Between Student-Perceived Teachers’ Responses and Students’ Bullying Behavior

To assess the associations between student-perceived teachers’ responses and both bullying measures, correlations were added to the final partial factor means invariance model. A model with freely estimated associations across the Italian and Belgian sample was compared to a model with associations that were restricted to be the same across the samples to investigate the invariance of the associations in the two samples. Regarding students’ self-reported bullying, the restricted model (χ^2^(236) = 373.60, CFI = 0.920, RMSEA = 0.050, SRMR = 0.102, BIC = 21,325) did not fit the data worse than the freely estimated model (χ^2^(230) = 370.59, CFI = 0.918, RMSEA = 0.051, SRMR = 0.099, BIC = 21,351; |ΔCFI| = 0.002, |ΔRMSEA| = 0.001, |ΔBIC| = 26 and lower in the restricted model). Hence, the relations between student-perceived teachers’ responses and self-reported bullying can be assumed the same across the Italian and Belgian sample. Self-reported bullying was positively associated with Non-Intervention, as 78% (Italian sample) and 77% (Belgian sample) of the coefficients were significant across the 100 datasets (Italian sample: average β = 0.18, average *SE* = 0.08; Belgian sample: average β = 0.13, average *SE* = 0.06). Although the associations were restricted to be equal across countries, separate values per sample were reported for the association with Non-Intervention, because of the released factor variance of Non-Intervention in the measurement invariance analyses. Further, self-reported bullying was negatively associated with Disciplinary Methods (average β = −0.18, average *SE* = 0.06, significant coefficients: 97%), Mediation (average β = −0.17, average *SE* = 0.06, significant coefficients: 98%), and Victim Support (average β = −0.18, average *SE* = 0.06, significant coefficients: 99%). Group Discussion did not show a significant association with self-reported bullying (average β = −0.03, average *SE* = 0.05, significant coefficients: 0%).

Regarding peer-nominated bullying, the restricted model (χ^2^(236) = 373.27, CFI = 0.922, RMSEA = 0.050, SRMR = 0.098, BIC = 19,546) did not fit the data worse than the freely estimated model (χ^2^(230) = 367.60, CFI = 0.922, RMSEA = 0.050, SRMR = 0.096, BIC = 19,569; |ΔCFI| = < 0.001, |ΔRMSEA| = < 0.001, |ΔBIC| = 23 and lower in the restricted model). Hence, the relations between student-perceived teachers’ responses and peer-nominated bullying can be assumed the same across the Italian and Belgian sample. No significant associations between peer-nominated bullying and Non-Intervention (Italian sample: average β = 0.03, average *SE* = 0.07, significant coefficients: 0%; Belgian sample: average β = 0.02, average *SE* = 0.05, significant coefficients: 0%), Disciplinary Methods (average β = −0.06, average *SE* = 0.05, significant coefficients: 7%), Group Discussion (average β = −0.05, average *SE* = 0.05, significant coefficients: 4%), Mediation (average β = −0.09, average *SE* = 0.05, significant coefficients: 36%), or Victim Support (average β = −0.09, average *SE* = 0.06, significant coefficients: 20%) were found. Thus, peer-nominated bullying was not associated with any of the teachers’ responses.

## Discussion

The aim of this study was to contribute to the research domain of teachers’ responses to bullying by validating the student-reported Teachers’ Responses to Bullying Questionnaire (TRBQ) in fourth and fifth grade of elementary school in the Italian and Belgian educational contexts. In line with our expectations, support for a five-factor structure was found, with Non-Intervention, Disciplinary Methods, Group Discussion, Mediation, and Victim Support as student-perceived teachers’ responses to bullying. This finding contributes to the understanding of teachers’ responses to bullying. Four active teachers’ responses can be distinguished: one focused on disciplining the bully, one focused on the (class) group, one focused on intermediation between the victimized student and the student(s) who bullied, and one focused on supporting the victimized student. Moreover, it is useful to consider Non-Intervention as a separate teachers’ response, and not merely as the absence of any of the active responses. This first research question has specifically given insight into the important perspective of younger students (i.e., elementary school students) and extended the use and validation of the TRBQ beyond the Italian educational context.

Second, the findings supported a partial factor means invariance model across the Italian and Belgian educational contexts. Hence, meaningful comparisons between the latent means across contexts can be made (Cheung and Rensvold, 2002). Moreover, finding (partial) scalar invariance is a prerequisite for substantive analyses (Hussey and Hughes, 2020), such as our investigation of the associations between student-perceived teachers’ responses and students’ bullying behavior. Some of the parameters were non-invariant across the Italian and Belgian students. First, the Non-Intervention factor explained more variance of one of its items (i.e., item 2) for Italian than Belgian students. Moreover, a higher Non-Intervention factor variance indicated a larger dispersion of the factor scores for the Belgian students compared to the Italian students. These findings could possibly be explained by the differential response rates and understanding of the Non-Intervention items. Namely, relatively more Belgian than Italian students answered the Non-Intervention items. However, during data collection it was discovered that they did not necessarily fully understand these items due to the double negotiation. Both the differential response rate and the understanding of the items could have led to a larger spread of scores for the individual item as well as the factor score for the Belgian students compared to the Italian students, many of whom did not answer these Non-Intervention items. Second, when comparing the latent means across the Italian and Belgian educational contexts, the latent mean of group discussion was higher for Italian students compared to Belgian students. Thus, Italian students perceived their teachers as using more Group Discussion responses than Belgian students perceived their teachers. This difference may be explained by the different educational contexts of Italy and Belgium. In 2017, a law on cyberbullying was introduced in Italy. It has a psycho-educational approach focused both on prevention and intervention and, among other statements, it obliged schools principals to appoint a teacher responsible for (cyber)bullying in each school. These teachers must be trained on the phenomena and a free online training on (cyber)bullying prevention and intervention strategies is provided by the Ministry of Education (Piattaforma Elisa, 2018). At the beginning of 2020/2021 school year, this 25 h training was fulfilled by more than 16,000 teachers responsible for (cyber)bullying: 1 out of 3 schools in Italy has the responsible teacher trained. Next to this law, more awareness for (cyber)bullying was raised by the training and other national projects promoted by the Ministry of Education (e.g., Generazioni Connesse, 2019) and bullying received increased attention within schools. Contrary, in Flanders (Belgium), schools are not obliged by law to have anti-bullying policies or teacher training in handling bullying. Therefore, it is possible that Italian teachers discuss bullying more frequently both among themselves and with their students. Furthermore, the use of Group Discussion is a method frequently reported by Italian teachers. More specifically, Campaert et al. (2017) found Group Discussion as the most prevalent teachers’ response in their study. In sum, promising first results for the validity of the TRBQ for use in upper elementary school in both Italy and Belgium were found. Future research could investigate the issues of the Non-Intervention scale. In addition, more research is needed to demonstrate the validity of the instrument across other countries.

With the third research question, we intended to contribute to the convergent validity of the TRBQ by examining the associations between student-perceived teachers’ responses and students’ bullying behavior. For self-reported bullying, associations with student-perceived teachers’ responses were found in the expected directions, which were similar to the findings of prior research (Campaert et al., 2017; Nappa et al., 2021). Non-Intervention was positively associated with self-reported bullying, indicating that more student-perceived Non-Intervention responses were related to more self-reported bullying. Most of the active teachers’ responses (i.e., Disciplinary Methods, Mediation, and Victim Support) were negatively associated with self-reported bullying, indicating that more student-perceived active teachers’ responses were related to less self-reported bullying. However, no association between Group Discussion and self-reported bullying was found. Possibly, the items measuring Group Discussion mostly reflect a single action and not a systematical approach to involve the class group in preventing and reducing bullying. Single group discussion actions may have a smaller impact on bullying. This is in line with the extensiveness of effective anti-bullying methods that involve the class group, such as the Support Group Method and the No Blame Approach (Rigby, 2014; van der Ploeg et al., 2016; Wachs et al., 2019).

For peer-nominated bullying, no associations with student-perceived teachers’ responses were found. Although peer-nominated measures are highly appreciated and used in social behavior research (van den Berg et al., 2015), they have limitations as well (e.g., Olweus, 2013). Specifically, the peer nominations used in our study can be considered as measures of behavioral reputation rather than the relational network reality (Stassen Berger, 2007; Veenstra and Huitsing, 2021). Behavioral reputations are often quite stable and changing peers’ opinions on a student’s reputation takes time (Davis and Lease, 2007); hence, it might be more difficult for teachers to affect peer nominations of bullying. In sum, we can conclude partial evidence for associations between bullying behavior and student-perceived teachers’ responses. Thus, the TRBQ seems to catch the dynamics between students and teachers regarding bullying from the individual student’s viewpoint.

### Limitations

A number of limitations of our study should be considered. First, the associations between student-perceived teachers’ responses and bullying measures were examined cross-sectionally. Therefore, conclusions about causal or temporal relations are not possible. Future longitudinal research can contribute to this study by examining the relationship between student-perceived teachers’ responses and bullying over time. Moreover, in addition to our innovative use of student-perceived teachers’ responses, a combination of both teacher- and student-reported teachers’ responses could benefit the research domain. In this way, it is possible to investigate the differences and similarities between the perceptions of teachers and students. Third, a variable-centered approach was used in this study, which might not represent the complexity of bullying situations and, therefore, the possible use of multiple teachers’ responses. In line with the study of Bayram Özdemir et al. (2021) it would be interesting to study the teachers’ responses to bullying with a person-centered approach, allowing for combinations of teachers’ responses. In this way, one acknowledges that teachers may respond differently to different bullying situations and that teachers may differ from each other regarding which (combination of) responses they apply. Fourth, although it is a strength that similar items were used to measure self-reported and peer-nominated bullying across the Italian and Belgian sample, it is a disadvantage that only one item was used to measure these constructs. On the other hand, these items are commonly-used in bullying research. Fifth, the internal consistency of the Non-Intervention scale was rather low and difficulties with this scale were discovered during data collection and data analyses. About a quarter of the Italian students did not answer any of the Non-Intervention items. Most Belgian students did answer the questions, but possibly not always with full understanding of the item and, therefore, not always reliable. The double negotiation in the Non-Intervention items and answer possibilities might be problematic, especially for elementary school children. The latter is supported by the finding of higher Cronbach’s alphas for the Non-Intervention scale in studies with middle and high school students (Campaert et al., 2017; Nappa et al., 2021). Therefore, future administrators of the TRBQ are advised to give sufficient written or verbal information about the double negotiation of the Non-Intervention items.

Despite these limitations, we have made a contribution to the emerging research domain of teachers’ responses to bullying with one of the first studies to validate elementary school students’ perceptions of teachers’ responses. We have found support for the five-factor structure of the TRBQ, with Non-Intervention, Disciplinary Methods, Group Discussion, Mediation, and Victim Support as student-perceived teachers’ responses. Furthermore, these teachers’ responses can be similarly measured in Italian and Belgian educational contexts and the latent means across these groups can be compared. Associations were found between self-reported, but not peer-nominated, bullying and most student-perceived teachers’ responses.

## Data Availability Statement

The datasets presented in this article are not readily available because the data sets generated and/or analyzed during the current study are available from the corresponding author on reasonable request. Requests to access the datasets should be directed to FG, fleur.vangils@kuleuven.be.

## Ethics Statement

The studies involving human participants were reviewed and approved by Social and Societal Ethics Committee (SMEC) KU Leuven. Written informed consent to participate in this study was provided by the participants’ legal guardian/next of kin.

## Author Contributions

FG conceived the study, coordinated the data collection, constructed the hypotheses, performed the statistical analyses, interpreted the results, and drafted the manuscript. HC and KV conceived the study, constructed the hypotheses, interpreted the results, and helped to draft the manuscript. KD and IB coordinated the data collection and gave feedback on the manuscript. EM conceived the study, constructed the hypotheses, interpreted the results, and gave feedback on the manuscript. BP conceived the study, constructed the hypotheses, interpreted the results, and helped to draft the manuscript. All authors read and approved the final manuscript.

## Conflict of Interest

The authors declare that the research was conducted in the absence of any commercial or financial relationships that could be construed as a potential conflict of interest.

## Publisher’s Note

All claims expressed in this article are solely those of the authors and do not necessarily represent those of their affiliated organizations, or those of the publisher, the editors and the reviewers. Any product that may be evaluated in this article, or claim that may be made by its manufacturer, is not guaranteed or endorsed by the publisher.

## Appendix

### Teachers’ Responses to Bullying Questionnaire

What does your teacher do when there is bullying in your class or at your school?

1. My teacher does nothing. ^a^
2. My teacher does not notice the bullying. ^a^
3. My teacher lets the students solve it alone. ^a^
4. My teacher helps the students involved to solve the bullying. ^b^
5. My teacher discusses bullying with the whole class. ^c^
6. My teacher discusses with the whole class how much the victim can suffer as a result of the bullying. ^c^
7. My teacher tries to get the students to make peace. ^b^
8. My teacher helps the (involved) students to find a solution for the bullying episode. ^b^
9. My teacher tries to have the victim consoled and helped by other students in the class. ^d^
10. My teacher tries to help the victim. ^d^
11. My teacher consoles the victim. ^d^
12. My teacher says to the bully/bullies that his/her/their behavior is unacceptable. ^e^
13. My teacher takes measures against the bully/bullies. ^e^
14. My teacher reports the bullying episode to the principal or to the parents. ^e^
15. My teacher explains what bullying is and discusses it with the class. ^c^

All questions are rated on a 5-point Likert scale: 1 = *Never*, 2 = *Almost never*, 3 = *Sometimes*, 4 = *Often*, 5 = *Always*.

The items belong to the following *a priori* scales: ^a^: Non-Intervention, ^b^: Mediation, ^c^: Group Discussion, ^d^: Victim Support, and ^e^: Disciplinary Methods.
